# Neuroprotective Effect of *Nypa fruticans* Wurmb by Suppressing TRPV1 Following Sciatic Nerve Crush Injury in a Rat

**DOI:** 10.3390/nu12092618

**Published:** 2020-08-27

**Authors:** Mi-Sun Kang, Gil-Hyun Lee, Go-Eun Choi, Hae-Gyung Yoon, Kyung-Yae Hyun

**Affiliations:** 1 Department of Rehabilitation Medicine, Pusan National University Yangsan Hospital, Yangsan 50612, Korea; misunpnuyh@gmail.com; 2 Department of Clinical Laboratory Science, Dong-Eui University, Busan 47340, Korea; rokmagnum@naver.com; 3 Department of Clinical Laboratory Science, College of Health Sciences, Catholic University of Pusan, Busan 46252, Korea; gechoi@cup.ac.kr; 4 Department of Art & Design, Dong-Eui University, Busan 47340, Korea; hgyoon@deu.ac.kr

**Keywords:** *Nypa fruticans* Wurmb (NF), sciatic nerve crush injury, neuroprotective, TRPV1, iNOS, NF-κB

## Abstract

Peripheral nerve injury can result in severe functional impairment and decreased quality of life due to loss of sensory and motor function. *Nypa fruticans* wurmb (NF) has been used in diverse folk remedies in East Asia. We have previously shown that *Nypa fruticans* wurmb extract has antinociceptive and anti-inflammatory effects by suppressing TRPV1 in the sciatic nerve injury. The present study investigated the effects of NF on the control of TRPV1 in relation to neuroprotective effects of a sciatic nerve crush injury. To evaluate the neuroprotective effects, an animal behavior test and a physiological function test were performed. Functional recovery and nerve recovery were improved in the NF and NF + SB (SB366791; TRPV1 antagonist) treated group. In the histomorphology evaluation, the neuronal regenerative effect of NF on the injured sciatic nerve was confirmed via hematoxylin and eosin (H&E) staining. In this study, the NF and NF + SB treated group showed neuroprotective and functional recovery effects from the sciatic nerve crush injury. Furthermore, the expression of NF-κB and iNOS showed a significantly suppressive effect on NF (*p* < 0.01), SB (*p* < 0.01), and NF + SB (*p* < 0.01) treated group at the 7th and 14th day compared to the vehicle group. This study confirmed the neuroprotective effects of NF on suppressing TRPV1 in a sciatic nerve crush injury. The findings of this study establish the effect of NF as a neurotherapeutic agent to protect the peripheral nerve after a sciatic nerve crush injury.

## 1. Introduction

A peripheral nerve injury presents a lifelong disability where crush injury is the highest rated type among other injuries [1]. Peripheral nerves are frequently exposed to physical injury, which can result in a severe functional impairment and decreased quality of life because of loss of sensory and motor function [2]. In the last decade, several therapeutic approaches have been developed to stimulate the regeneration of the nerve, such as the administration of neurotrophins [3,4,5] or extracellular matrix molecules [6,7,8,9], or the application of electrical stimulation [8,9]. Unfortunately, all these methods are limited in their scope because they do not ensure a full restoration of function. For this reason, new strategies that simultaneously potentiate axonal regeneration while promoting remyelination and the recovery of nerve functions are needed. Experimentally, many different types of medications have been used in rat crush injury models, such as steroids, nonsteroidal anti-inflammatory drugs, and vitamins [10,11,12]. Among other therapeutic strategies, pharmacotherapy has been shown to be a promising approach to neurorehabilitation, with the exploration of potential natural products that can improve the efficacy of nerve regeneration attracting considerable research interest [13]. Several plants and plant-derived compounds have been identified to accelerate the recovery process after peripheral nerve injury [14]. Recently, *N.fruticans* Wurmb (NF), which was used as a folk remedy, is a plant that is getting more attention because of its various effects. *N.fruticans* Wurmb, which belongs to the Arecaceae family, is a mangrove plant that grows on the surface of mudflats and salt marshes in East Asia [15]. In traditional medicine, leaves, stem, and roots of NF are used to treat asthma, leprosy, tuberculosis, sore throat, liver disease, snake bite, and as a pain reliever, and can also be used as sedative and carminative [16,17]. NF is known to be rich a variety of compounds such as polyphenols and flavonoids [15,18]. NF has been reported to exert various biological activities including antihyperglycemic, antinociceptive, antidiabetes, and antioxidant effects [19,20]. Several researches have also shown that using anti-inflammatory agents can support nerve regeneration and myelination [21,22,23]. Furthermore, other studies have demonstrated that ion channel inhibition including TRPV1 can promote axon regeneration [24,25]. Our previous study showed antinociceptive and anti-inflammatory effects of NF in cases of sciatic nerve injury by suppressing TRPV1 [26]. However, the neuroprotective effect of NF on peripheral nerve crush injury in rats has been rarely studied, particularly in relation with TRPV1. Therefore, this study was carried out with the hypothesis that the neuroprotective effects of NF by suppressing TRPV1 on a sciatic nerve crush injury model.

## 2. Materials and Methods

### 2.1. Experimental Animals

Male Sprague-Dawley rats (Hyochang science, Daegu, Korea) weighing 100–120 g, aged 4 weeks were used in this study. The animals were kept at a constant ambient temperature of 20 ± 2 °C and the humidity was maintained at 55 ± 5% with a 12/12-hr light/dark cycle with sufficient water and feed. Experiments were carried out after a one week adaptation period. All experimental procedures were performed following the approval of the Animal Experiment Ethics Committee of Dong-Eui University (R2018-002).

### 2.2. Surgical Procedure (Sciatic Nerve Crush Model, SNC)

All surgical procedures were performed under general anesthesia with 300 mg/kg of Tribromoethanol (Avertin) administered intraperitoneally. The sciatic nerve was exposed by making a 2 cm skin incision in the right posterior femur, minimizing the damage to the surrounding tissues. In the exposed sciatic nerve, hemostatic forceps were used to crush the sciatic nerve for 30 s according to the modified procedure described by Kalender et al. [27]. The skin was sutured with 4-0 stitches, and was sterilized to prevent infection.

### 2.3. Extraction of Nypa Fruticans Wurmbs

This experiment used dried flower stalk of *Nypa fruticans* Wurmb (Todipalm Korea; Hanam City, Korea) imported from Myanmar. Pulverized NF powder was mixed with 80% EtOH (*v*/*v*) at a ratio of 1:10 at room temperature for 4 h. After the extraction, the mixture was filtered and the filtered liquid components were put in a rotatory evaporator to prepare a pure extract. The concentrated liquid was lyophilized and stored at −20 ℃ in sterile universal bottles. The extract yield obtained was 4.7%.

### 2.4. Treatments

**Figure 1:**
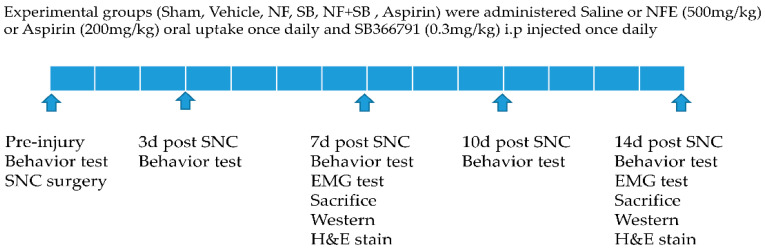
Experimental design and protocol for the drug application.

The rats were randomly divided into six groups. A scheme of the protocol followed is shown in Figure 1. Experimental groups were the sham group (Intact, saline 10 mL/kg), in which animals did not undergo any surgical procedures; the vehicle group (SNC, saline 10 mL/kg), which received the sciatic nerve crush (SNC) injury and were treated with physiological saline; the NF group (SNC, NFE 500 mg/kg), which received the crush injury and were treated with NFE 500 mg/kg oral uptake once daily after surgery; and, the aspirin group (SNC, 200 mg/kg), which received the crush injury and were treated with aspirin (200 mg/kg) through oral uptake once daily after surgery. Aspirin (Bayer, Inc., Hanover, NJ, USA) as a positive control was dissolved in physiological saline. The SB group (SNC, SB366791 0.3 mg/kg) which received the crush injury were intraperitoneally injected once daily at 0.3 mg/kg. The SB-366791 (Enzo Life Sciences, Farmingdale, NY, USA) as a TRPV1 antagonist was purchased from Enzo (Farmingdale, NY, USA), dissolved in 100% ethanol (10 mg/mL with warming) and diluted in physiological saline. SB366791 [N-(3-methoxyphenyl)-4-chlorocinnamide] is a more selective and in vivo is also a more potent TRPV1 antagonist than the commonly-used TRPV1 antagonist capsazepine [28], and has been widely used as a selective TRPV1 antagonist in pain research [29,30,31]. The SB + NF group (SNC, NFE 500 mg/kg + SB366791 0.3 mg/kg) which received the crush injury were intraperitoneally injected once daily at 0.3 mg/kg, with NFE 500 mg/kg given via oral uptake once daily after surgery.

### 2.5. Functional Analysis

#### 2.5.1. Rotarod Measurement

To assess motor deficits and balance in mice with a sciatic nerve crush injury, it was measured using a rotarod device. This is made up of four cubicles with a diameter of 7 cm and a gap of 15 cm, and cylindrical rods capable of rotating at a height of 60 cm. Two training sessions were carried out at 5 min each for 3 days before analysis. When the rotarod was measured, the experimental rat was placed on a rotating rod, and the time taken to gradually fall up to a maximum of 300 s was measured by gradually increasing the speed (2–20 rpm). All animals were examined three times at pre- injury and on the 3rd, 7th, 10th, and 14th day after injury, and the mean value was measured as walking time.

#### 2.5.2. Walking Track Analysis

To measure motor nerve recovery, experimental groups were measured pre-injury and on the 3rd, 7th, 10th, and 14th day after injury. The hind feet were dipped in blue ink and footprints were recorded on white paper while walking through a passage 80 cm long, 7 cm wide, and 10 cm above on the floor. At the end of the pathway, a dark room was made and the animals were allowed to move forward. This was repeated three times, and the footprint was measured. The sciatic functional index (SFI), which is an evaluation measure of motor function, shows a value close to 0 in the normal state and closer to −100 with complete injury of the sciatic nerve. The sciatic functional index (SFI) was calculated using the method proposed by Bain et al. [32].

### 2.6. Electrophysiological Testing

The sciatic nerve was measured by electromyogram (Keypoint, Minneapolis, MN, USA) on the 7th and 14th day after the nerve crush injury. After exposing the sciatic nerve of anesthetized rats, bipolar hooked platinum stimulation was applied to the distal and proximal portions of the injured sciatic nerve (stimulus duration 0.04 ms) and bipolar recording electrodes were recorded in the gastrocnemius. (high frequency filter 10,000 Hz, low frequency filter 20 Hz, gain 5 mV/D, sweep 1 ms/D) with a stimulus of 1–8 mA. The latency and the peak amplitude of compound motor action potentials (CMAP) were obtained. The length of the sciatic nerve between the two electrodes was measured and the length was divided by the time taken for conduction to determine the nerve conduction velocity (NCV). The same electromyogram was performed on the normal side.

### 2.7. Hematoxylin–Eosin Staining

The sciatic nerve was fixed with 4% paraformaldehyde on the 7th day and the 14th day after the sciatic nerve crush injury. Paraffin sections of 5 µm were prepared. The sections were stained with hematoxylin solution for 5 min and Eosin solution for 3 min. Changes in the sciatic nerve were observed using an optical microscope (Olympus BX50, Olympus Optical Co., Tokyo, Japan).

### 2.8. Western Blotting Analysis

Expressions of NF-κB and iNOS in the sciatic nerve samples of the experimental groups at the 7th day and the 14th day were detected using western blotting analysis. A primary rabbit polyclonal NF-κB p65 (1:1000, Cell Signaling, Technology Inc., Denvers, CO, USA) and iNOS (1:1000, Cell Signaling, Technology Inc., Denvers, CO, USA) Ab were reacted with the horseradish peroxidase-conjugated secondary Ab (Cell Signaling, Technology Inc., Denvers, USA) at dilutions of 1:1000, respectively. Relative expression levels of all proteins were determined through densitometry and normalized by actin.

### 2.9. Statistical Analysis

All data from the studies was expressed as means ± SEM. Version 18 of the SPSS software package was used for data analysis. Significance was determined using ANOVA followed by Tukey test as the post-hoc tests and Kruskal–Wallis test with Dunn’s multiple comparison test. *p* values less than 0.05 indicated statistical significance.

## 3. Results

### 3.1. NF Administration Improved Functional Recovery of the Sciatic Nerve on Walking Track Test

**Figure 2:**
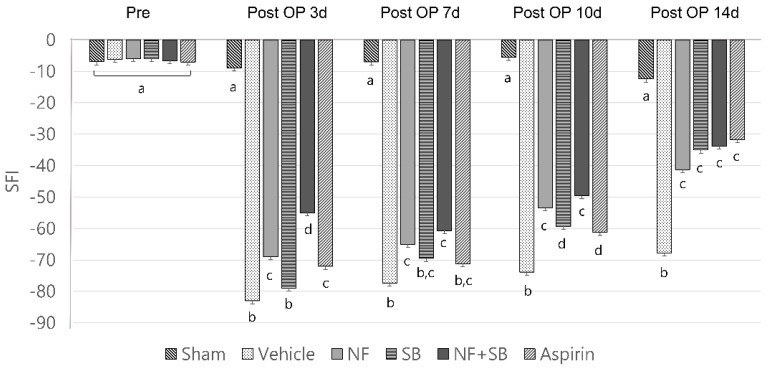
Effect of NF on walking track test at 3rd, 7th, 10th, and 14th day after crush nerve injury. The NF treated group showed recovery of gait function. Levels of SFI (sciatic function index) were measured based on the footprints on the walking track. Data are expressed as mean SFI ± SD. Data were analyzed by ANOVA with Tukey’s post hoc analyses. Values with the different small letters (a–d) are significantly different among six treated groups of same time group at *p* < 0.05.

A number of experimental injuries and treatments have shown that SFI is a significantly useful tool for the evaluation of functional recovery of the sciatic nerve of rats [33]. Walking ability was measured in order to determine the effect of the NF, SB, and NF + SB treatment on functional recovery after sciatic nerve injury. Evaluation of SFI was performed pre-injury and on the 3rd, 7th, 10th, and 14th day after sciatic crush nerve injury. In the walking track, the NF group (−68.9 ± 3.7, −65 ± 5.1, −53 ± 1.6, −41 ± 5.0; *p* < 0.05), SB group (−78.9 ± 2.8, −69.5 ± 5.5, −59.3 ± 2.0, −35 ± 6.7; *p* < 0.05), and NF + SB group (−75.8 ± 3.7, −69.1 ± 2.8, −55.6 ± 1.6, −43.1 ± 5.4; *p* < 0.05) showed significant differences compared to the vehicle group (−82.9 ± 5.8, −77.3 ± 6.6, −73.8 ± 3.0, −55.1 ± 3.6) in SFI values on the 3rd, 7th, 10th, and 14th day after injury (Figure 2). This implies that the NF in the sciatic nerve injury affects the recovery of gait function. Also, the SB and NF + SB treated group showed a recovery of gait function similar to the NF treated group.

### 3.2. NF Administration Improved Functional Recovery of the Sciatic Nerve on Rotarod Test

**Figure 3:**
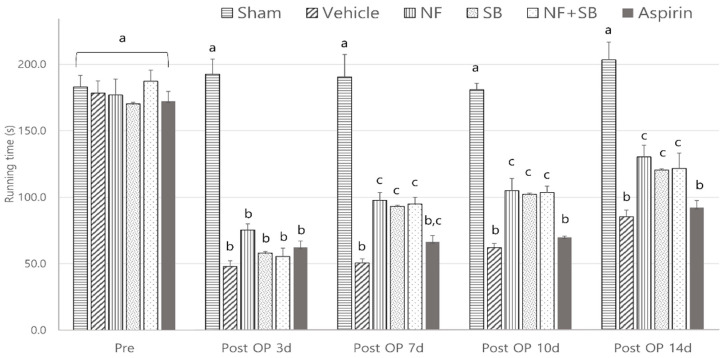
Effect of NF on Rotarod test at the 7th, 10th, and 14th day after the nerve crush injury. The NF treated group showed recovery of motor deficit and balance with increased riding time. Results are represented as mean ± SEM. Data were analyzed by ANOVA with Tukey’s post hoc analyses. Values with the different small letters (a–c) are significantly different among six treated groups within same time group at *p* < 0.05.

The rotarod test using the rotarod device evaluated exercise deficit and balance. We can evaluate motor and sensory input as measures of coordination on the rotarod. In the rotarod test, the NF group (97 ± 20, 104 ± 32, 130 ± 30; *p* < 0.05), SB group (93 ± 10, 102 ± 19, 120 ± 36; *p* < 0.05), and NF + SB group (95 ± 17, 103 ± 17, 121 ± 40; *p* < 0.05) showed significant differences compared to the vehicle group (50 ± 11, 62 ± 11, 85 ± 17) in total riding time at the 7th, 10th, and 14th day (Figure 3).

### 3.3. NF Administration Improved Nerve Recovery of the Sciatic Nerve on the Electrophysilogical Measurements

**Figure 4:**
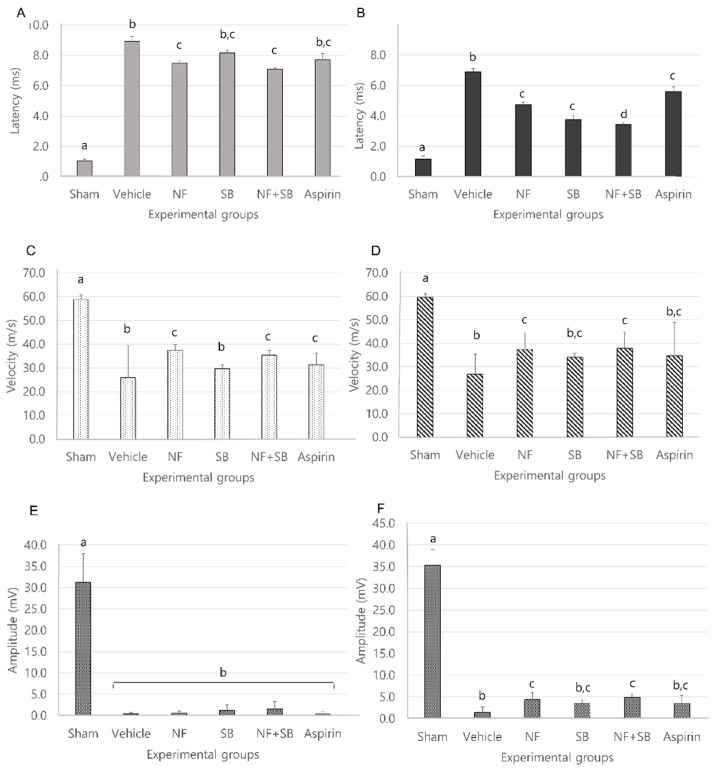
Effect of NF and NF + SB at the 7th and 14th day after nerve crush injury with latency, nerve conduction velocity and amplitude of compound action potential in each group. Latency of each group at 7th (**A**) and 14th day (**B**); NCV of each group at 7th (**C**) and 14th day (**D**); Amplitude of each group at 7th (**E**) and 14th (**F**) day. Results are represented as mean ± SEM. Data were analyzed by ANOVA with Tukey’s post hoc analyses; Values with the different small letters (a–c) are significantly different among six treated groups at *p* < 0.05.

Electromyography (EMG) indicated that regenerated nerve fibers had successfully reinnervated with the gastrocnemius muscle (GCM). Nerve conductive velocity (NCV) showed remyelinated nerve fiber conditions and amplitude reflected regenerative axon nerve fiber condition. Nerve conduction study (NCS), Latency, CMAP amplitude were conducted on the control group and the experimental group at the 7th and 14th day after sciatic nerve crush injury. In the EMG test, it was confirmed that latency values were decreased in the group treated with NF and NF + SB at the 7th day (Figure 4A) and NF, SB, and NF + SB on the 14th day (Figure 4B) after the sciatic nerve crush injury. The nerve conduction velocity (NCV) values were increased in the nerve conduction study in the group treated with NF and NF + SB on the 7th (Figure 4C) and the 14th day (Figure 4D) after the sciatic nerve crush injury. Moreover, CMAP amplitude was increased in the nerve conduction study in the groups treated with NF and with NF + SB on the 14th day (Figure 4F) after the sciatic nerve crush injury compared to the vehicle group. NF and NF + SB treatment indicated that more regenerative axons and reinnervated GCM, which might also lead to a significantly higher CMAP amplitude (*p* < 0.05) in comparison with that of the vehicle group.

### 3.4. NF Administration Improved Histopathological Changes in the Sciatic Nerve Crush Injury Models

**Figure 5:**
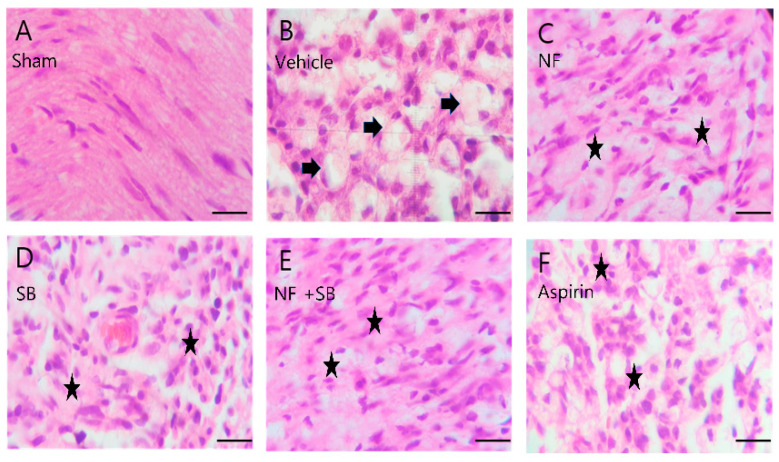
Representative H&E staining of sciatic nerves on 7th day after surgery. (**A**) Sham group shows well-organized sciatic nerve fibers and no structure defect shows well-organized nerve fiber tissue. (**B**) Vehicle group shows several areas of edema and degraded myelin sheath with vacuolation (arrows). (**C**) NF group, (**D**) SB group, (**E**) NF + SB group, (**F**) aspirin group, (**C**–**F**) The nerve fiber density was increased, and the vacuolar-like degeneration was decreased at the 7th day (asterisks). Scale bars = 10 µm.

**Figure 6:**
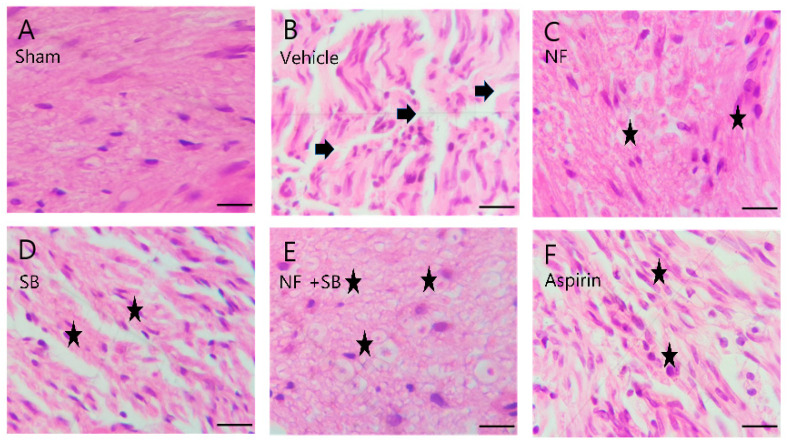
Representative H&E staining of sciatic nerves on 14th day after surgery. (**A**) Sham group shows well-organized sciatic nerve fibers and no structure defects. (**B**) Vehicle group shows several areas of edema and degraded myelin sheath (arrows). (**C**) NF group, (**D**) SB group, (**E**) NF + SB group, (**F**) aspirin group, (**C**–**F**) The nerve fiber density was increased and the vacuolar-like degeneration was decreased at the 14th day (asterisks). Scale bars = 10 µm.

Histologic evaluation was performed to evaluate the protective effect on the injured sciatic nerve via H&E stain. Observation was performed in the transverse sections of the midsection in each group. In the sham group (Figure 5A and Figure 6A), the sciatic nerve section showed a normal structure and architecture and no inflammation. In the vehicle group (Figure 5B and Figure 6B), the nerve fiber structure was not uniform and the density of the myelinated nerve fiber was decreased and exhibited contracted or vacuolar shapes. However, in the NF group (Figure 5C and Figure 6C), SB group (Figure 5D and Figure 6D), NF + SB group (Figure 5E and Figure 6E), and Aspirin group (Figure 5F and Figure 6F), the nerve fiber density increased and the vacuolar-like degeneration decreased at the 7th and 14th day after injury compared to the vehicle group. In particular, the NF and NF + SB treated group showed decreased areas of edema, and the nerve fibers seemed to be better organized than in the vehicle group.

### 3.5. NF Downregulates the Expression of iNOS in the Sciatic Nerve Crush Injury Models

**Figure 7:**
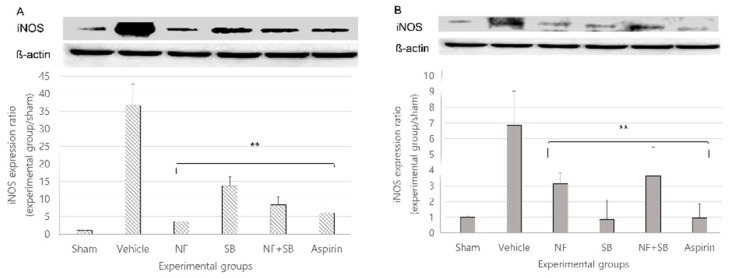
NF downregulates the expression of iNOS in the sciatic nerve (**A**,**B**) at the 7th and 14th day following the nerve crush injury. The density data are the mean ± SEM values of experiments. (** *p* < 0.01 vs. vehicle group, Kruskal–Wallis test followed by a post hoc Mann–Whitney U test).

The toxic free radical nitric oxide (NO), produced by induced nitric oxide synthase (iNOS) in macrophages, and participates in an early non-specific immunological reaction associated with cytotoxicity [34]. Peripheral nerve injury is also associated with local upregulation of iNOS in macrophages and Schwann cells [35,36,37] with subsequent NO release. At the 7th and 14th day after surgery, a sciatic nerve sampling was performed for western blot analysis. In the vehicle group, iNOS expression was increased compared to the sham group at the 7th and 14th day. However, the expression of iNOS showed a significantly suppressive effect in the NF (*p* < 0.01), SB (*p* < 0.01), and NF + SB (*p* < 0.01) treated groups at the 7th (Figure 7A) and 14th day (Figure 7B) compared to the vehicle group.

### 3.6. NF Downregulates the Expression of NF-ΚB in the Sciatic Nerve Crush Injury Models

**Figure 8:**
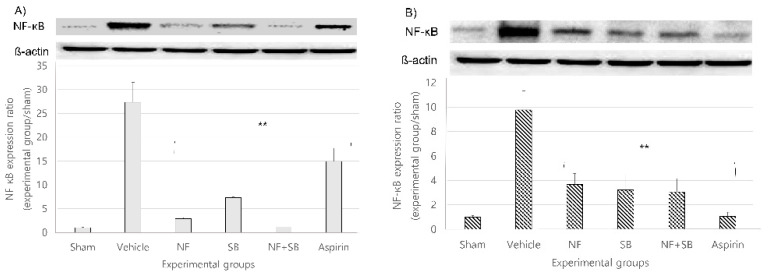
NF downregulates the expression of NF-κB in the sciatic nerve at the 7th (**A**) and 14th (**B**) day following the crush nerve injury. The density data are the mean ± SEM values of experiments. (** *p* < 0.01 vs. vehicle group, Kruskal–Wallis test followed by a post hoc Mann–Whitney U test).

NF-κB is a family of ubiquitously expressed, eukaryotic transcription factors that participate in the regulation of multiple immediate genes expressed at the onset of many vital biological processes, such as cell growth, immunoregulation, apoptosis, and inflammation [38,39]. The importance of the role of NF-κB in initiating a potent inflammatory response cannot be better signified than recognizing that the B consensus sequence is found in the promoter/enhancer regions of more than 50 diverse genes whose expression is known to be crucial in driving an inflammatory response [40,41,42]. At the 7th and 14th day after injury, a western blot analysis was performed on the sciatic nerve. In the vehicle group, NF-κB expression increased compared to the sham group at the 7th and 14th day. However, the expression of NF-κB showed a significantly suppressive effect in the NF (*p* < 0.01), SB (*p* < 0.01), and NF + SB (*p* < 0.01) treated group at the 7th (Figure 8A) and 14th (Figure 8B) day compared to the vehicle group.

## 4. Discussion

Nerve injury is known to alter the expression of various ion channels, including the TRPV1 channel [43]. Recent reports indicate that functional TRPV1 is expressed in the sciatic nerve and DRGNs [44,45]. Activation of TRPV1 enhanced Ca2+ accumulation due to its permeability to Ca2+ [45,46] which is involved in several physiological and pathological processes such as neuronal viability, apoptosis, and neuronal recovering signaling [47]. Several studies have reported that expression levels of TRPV1 are increased in the sciatic nerve and DRGN by sciatic nerve injury [48,49,50]. The validation of the TRPV1 channel as a therapeutic target for the control of pain and inflammatory conditions in a variety of diseases and injury states, has prompted the development of several TRPV1 agonists and antagonists that have entered clinical trials [51]. Our previous study demonstrated that the NF and NF + SB treated group showed decreased pro-inflammatory cytokines levels and inflammatory expression factor, COX2 protein expression in the sciatic nerve crush injury through the suppression of TRPV1. These results support the premise that NF affects TRPV1 inhibition, and consequently, has anti-inflammatory and antinociceptive effects. A follow-up study was carried out with the hypothesis that NF has neuroprotective effects by suppressing TRPV1 on a sciatic nerve crush injury model. The rat sciatic nerve model is widely used for simultaneous evaluation of motor and sensory nerve function after nerve injury [33,52,53,54]. This study conducted with validated in vivo models. Walking track analysis, rotarod, and EMG test have become a popular method of assessing functional recovery and nerve regeneration from a nerve injury [55,56,57,58,59]. Walking track analysis is the gold standard for evaluation of nerve recovery after sciatic nerve injury because proper walking requires coordinated function involving sensory input, motor response, and cortical integration. It is a useful technique if the research focus is on the functional outcome [10]. In the walking track (Figure 2), the NF, SB, and NF + SB treated group showed significant differences compared to the vehicle group in SFI values at the 3rd, 7th, 10th, and 14th day after injury. Also, we evaluated motor and sensory input as measures coordination on the rotarod (Figure 3). In the NF, SB, and NF + SB treated groups, total riding time is significantly increased compared to the vehicle group at the 7th, 10th, and 14th day after nerve crush injury. Furthermore, an electrophysiology study was performed to further investigate the motor functional recovery. The CMAP amplitude is directly proportional to the number of nerve fibers innervating the target muscle, which allows a further evaluation of motor functions [60]. In the EMG test, it was confirmed that latency values were decreased in the group treated with NF and NF + SB at the 7th day, and in the group treated with NF, SB, and NF + SB at the 14th day after the sciatic nerve crush injury (Figure 4A,B). CMAP amplitude was increased in the nerve conduction study in the group treated with NF and NF + SB at the 14th day after sciatic nerve crush injury compared to the vehicle group (Figure 4E,F). Furthermore, a histomorphologic evaluation confirmed the neuronal protective effect of NF in the injured sciatic nerve via H&E stain (Figure 5 and Figure 6). The NF, SB, and NF + SB treated groups showed decreased areas of edema, and their nerve fibers seem to be better organized than the vehicle group at the 7th and 14th day. Consistently, these results demonstrated that NF shows beneficial effects in inducing neuronal and functional nerve recovery.

Following peripheral nerve damage, a series of well-described molecular and anatomical events, including Wallerian degeneration of the distal nerve stump and re-growth of axons, leads to whole or partial recovery of nerve function. Among the early nerve-centered inflammatory events are increased local levels of TNF-α and activated NF-κB [61,62,63,64]. As has been demonstrated with other neuropathological processes, inflammation appears to exert an inhibitory effect on axonal regeneration following nerve damage [65]. NF-κB is a well-known transcription factor in cells and is involved in multiple interactions such as oxidative stress, inflammation, and regulation of key pain-related molecules [66,67,68]. Our previous study showed that TRPV1 expression is suppressed in the NF treated group with a sciatic nerve crush injury at the 7th and 14th days with anti-inflammatory effect [26]. In previous reports, it has been demonstrated that induction of a sciatic nerve injury elevates oxidative stress levels in neurons [69,70] and as a consequence of excessive Ca2+ influx, apoptosis is caused by activation of cation channels [69]. It is known that oxidative stress is one of the main causes of nerve damage after injury [71]. Furthermore, TRPV1 could regulate the expression of iNOS at the gene level in macrophages through interference with upstream signaling events [72]. This means that iNOS expression can be regulated through TRPV1 inhibition. Also, in addition, our current study confirmed that NF-κB (Figure 8), a factor associated with apoptosis and a factor of generation of NO, iNOS (Figure 7) expression is downregulated in the NF and NF + SB treated group. Notably, our results further showed that NF modulated NF-κB p65 factor expression and exerted suppressive effects on iNOS, which decreased the cell damage caused by a nerve crush injury. We speculated that NF is suppressing of TRPV1, has might be mediated the regulation of NF-kb p65, and the inhibition of iNOS signals might be, at least in part, involved in the underlying mechanisms. Recently, previous study reported there are three transcription-binding sites for NF-κB p65 in the TRPV1 promoter region, suggesting that NF-κB p65 may also affect the expression of TRPV1 through transcriptional regulation [73]. However, we have limited study about NF-κB pathway related TRPV1 expression and further investigation is required the underlying mechanism.

All of the in vivo and in vitro evidence indicates the improving functional recovery of a nerve crush injury, and suppression of TRPV1 by NF may promote a nerve protective effect after nerve injuries. However, there was no significant difference in the in vivo and in vitro experiments compared to the group treated with NF alone and the group treated with NF + SB. Our data does not demonstrate any synergistic effects of the NF and TRPV1 antagonist.

## 5. Conclusions

In summary, NF may exert a neuroprotective effect by controlling the neurological function of TRPV1. The findings of the present study highlight the therapeutic benefits of NF as a functional recovery and neuroprotective agent for peripheral nerve injury through suppressing TRPV1. As the mechanism of NF’s neural protective effects is still unknown, further studies are required to explore the contribution of potential applications of NF in therapeutic management after a peripheral nerve injury.
